# Efficacy and adverse reactions of peripheral add multifocal soft contact lenses in childhood myopia: a meta-analysis

**DOI:** 10.1186/s12886-024-03408-7

**Published:** 2024-04-16

**Authors:** Desheng Song, Wen Qiu, Ting Jiang, Zhijun Chen, Juan Chen

**Affiliations:** 1 Department of Ophthalmology, Children’s Hospital of Nanjing Medical University, 72 Guangzhou Road, Gulou District, 210008 Nanjing, Jiangsu Province China; 2 Department of Nursing, Children’s Hospital of Nanjing Medical University, 210008 Nanjing, Jiangsu Province China

**Keywords:** Myopia, Soft contact lens, Children, Meta-analysis

## Abstract

**Objectives:**

This study aims to compare the efficacy of peripheral add multifocal soft contact lenses (SCLs) (excluding bifocal SCLs) with single vision contact lenses or spectacles in controlling myopia progression.

**Method:**

A comprehensive literature search was conducted in the Pubmed, EMBASE, Web of Science, and Cochrane Library databases until October 2023. The literature was thoroughly screened based on predetermined eligibility criteria. Pooled odds ratios (ORs) were calculated for dichotomous data and weighted mean differences (WMD) for continuous data.

**Results:**

A total of 11 articles comprising 787 participants were included in this meta-analysis. Our pooled results demonstrated that the peripheral add multifocal SCLs groups exhibited significantly reduced refraction progression (MD = 0.20; 95%CI, 0.14 ∼ 0.27; *P*<0.001) and less axial length elongation (MD=-0.08; 95%CI, -0.09∼-0.08; *P*<0.001) compared to the control group. There was no significant difference in high-contrast logMAR distance visual acuity between the two groups (MD = 0.01; 95%CI, -0.00 ∼ 0.02; *P* = 0.19). However, the group using single-vision lenses had better low-contrast logMAR distance visual acuity compared to those using peripheral add multifocal SCLs (MD = 0.06; 95%CI, 0.02 ∼ 0.10; *P* = 0.004). Data synthesis using a random-effects model indicated an incidence of contact lens-related adverse events of 0.065 (95%CI, 0.048 ∼ 0.083).

**Conclusions:**

The present meta-analysis signifies that peripheral defocus modifying contact lenses are effective in slowing down the progression of myopia and reducing axial elongation.

**Supplementary Information:**

The online version contains supplementary material available at 10.1186/s12886-024-03408-7.

## Background

Myopia is a prevalent ocular condition that predominantly affects children and adolescents, particularly in East Asia. Its global prevalence has witnessed a significant surge over recent decades, emerging as a global pandemic [1, 2]. Myopia, particularly progressive high myopia, poses a substantial risk factor for myopic choroidal neovascularization, cataracts, open-angle glaucoma, myopic macular degeneration, and rhegmatogenous retinal detachment [3]. These complications can lead to irreversible visual impairment or loss later in life. Consequently, clinicians, researchers, and practitioners are eagerly seeking an effective intervention to decelerate, halt, or even reverse myopia progression in children and adolescents.

Numerous strategies have been implemented to control myopia progression, such as atropine use, overnight corneal reshaping contact lenses (orthokeratology), peripheral defocusing spectacle lenses, limiting near work, and promoting outdoor activities. Peripheral add multifocal soft contact lenses, notable for their specialized optical design, have attracted considerable attention as an innovative method for controlling myopia. Research on the effectiveness of these lenses in controlling myopia progression has produced varied results. For instance, an 18-month study conducted by Lina et al. demonstrated that children wearing proclear multifocal SCLs experienced a 72% reduction in myopia progression and an 80% decrease in axial elongation compared to those using single-vision soft contact lenses (SV SCLs) over one year [4]. A 1-year Randomized Clinical Trial revealed that children wearing Menicon low-addition multifocal SCLs exhibited 47% less myopia progression and 26% less axial elongation than those using SV SCLs [5]. However, Sankaridurg et al. demonstrated that the myopia progression was merely slowed by 23% and axial elongation by 21% over two years using a novel central and peripheral plus contact lenses [6].

A previous meta-analysis evaluated the overall impact of concentric bifocal and peripheral add multifocal SCLs on retarding myopia progression, indicating that both concentric bifocal and peripheral add multifocal SCLs were associated with reduced myopia progression and decreased axial elongation [7]. However, that analysis had several shortcomings, including a limited number of studies, the inclusion of both concentric ring bifocal and peripheral add multifocal SCLs, a lack of high- and low-contrast visual acuity and soft contact lens-related adverse effects assessment, and reliance solely on changes in refraction and axial length during the first year of follow-up as the primary outcome measures for comparison.

This meta-analysis aims to thoroughly assess the overall effectiveness of peripheral add multifocal SCLs (excluding bifocal SCLs) in slowing myopia progression among children, with the mean annual changes in refraction and axial length as the primary outcome measures. It also includes a quantitative evaluation of high- and low-contrast visual acuity and soft contact lens-related adverse effects.

## Materials and methods

### Search strategy

The present meta-analysis employed Cochrane Review Methods. Two researchers (DSS and WQ) independently conducted comprehensive searches of the following electronic databases until October 2023: Web of Science, Medline, Embase, and the Cochrane Register of Controlled Trials databases. The search strategy was as follows: ((short OR near*) AND sight* OR myop*) AND (“contact lens*”) AND (refract* OR accommodation, ocular OR visual acuity OR (accommodat* or acuity) OR (progress* or slow* or retard* or funct*)) AND (clinical trial OR placebo). No language restrictions were applied, and for non-English articles, English abstracts were utilized. Additionally, the reference lists of all identified articles were manually searched for any additional relevant publications.

### Study inclusion and exclusion criteria

Controlled studies meeting the following inclusion criteria were considered (based on the ‘PICOS (Population, Intervention, Comparison, Outcome, Study design)’ principle)): (a) Population: Myopic children and adolescents aged between 6 and 18. (b) Intervention: Studies that investigated the efficacy of peripheral add multifocal SCLs for myopia control. (c) Comparison: Studies that included age, gender and refractive error matched control group. (d) Outcome: Studies that compared the changes in spherical equivalent refraction (SER) and axis length (AL) between the intervention and control groups lasted at least 12 months. (e) Study design: randomized controlled trial (RCT), historical controls, prospective and longitudinal studies. Studies were excluded based on two exclusion criteria: (1) letters, correspondence, and reviews; (2) unpublished articles (e.g., conference abstracts), case reports, or case series lacking a control group.

### Study selection and data extraction

Two investigators, DS Song and T Jiang, independently retrieved and screened the literature. Data provided in the articles were extracted using a literature data extraction table.

If there was any disagreement, a third author (J Chen) settled the discrepancy. Information extracted from the included literature encompassed the first author, publication year, length of follow-up, age, plus power, study design, sample size, treatment lens, baseline refraction, myopia control rate and axial length control rate. Statistical analysis was conducted using data from the final visit. This meta-analysis adhered to the PRISMA (Preferred Reporting Items for Systematic Reviews and Meta-Analyses) guidelines [8].

### Quality assessment

The quality of the included RCTs was assessed using the Cochrane evidence-based medicine system [9]. Each trial’s quality assessment encompassed five domains: Election bias (randomization order generation, allocation hiding); implementation bias (blind method); measurement bias (blind method in outcome evaluation); loss to follow-up bias (incompletely resolved data); publication bias (selective reporting of research results); and other biases. Each research was evaluated at “low risk of bias,” “high risk of bias,” or “unclear risk of bias.” Discrepancies were resolved by discussion. To assess the quality of nonrandomized cohort studies, the CASP (Critical Appraisal Skills Programme) scale was utilized. The scale evaluated the study quality by considering internal and external validity and posed predefined questions regarding data collection methods, study design, confounding, selection bias, dropout, and intervention integrity.

### Quality of evidence assessment

The quality of cumulative evidence derived from current meta-analyses was assessed using the GRADE approach. This assessment evaluated the confidence in the treatment-effect estimate (pooled MD) and was categorized as high, moderate, low, or very low level. Initially, evidence from an RCT dataset was considered high-quality, but it could be downgraded if the dataset exhibited certain concerns. These concerns included a high risk of bias, high inconsistency (considerable heterogeneity), indirectness (not obtained from a direct group), imprecision (a non-significant result), and high or indeterminable publication bias. In this study, the presence of each concern would result in a one-level downgrade of evidence quality. Conversely, evidence from an observational dataset was initially rated as low-quality, but it could be upgraded if the dataset exhibited certain characteristics. These characteristics included a very large effect value, a dose-effect relationship, and an absence of negative bias.

### Statistical analysis

Statistical analysis was performed using Review Manager 5.3 (Copenhagen: The Nordic Cochrane Centre, The Cochrane Collaboration, 2008). Pooled odds ratios (ORs) were calculated for dichotomous data, while weighted mean differences (WMD) for continuous data. Heterogeneity between studies was assessed using I^2^ and Q tests [10]. The fixed effect model was applied when *P*>0.05 or I^2^<50%, whereas the random-effect model was employed otherwise. Pooled results with p-values < 0.05 were considered statistically significant. Sensitivity analysis were conducted for each outcome by excluding each included study. Publication bias was assessed using Egger’s test. Subgroup analysis was performed if appropriate. The treatments were ranked based on their relative therapeutic effects compared to SV contact lenses or spectacles. A “strong” effect was defined when the one-year change in refraction was ≥ 0.50D or the axial length change was ≥ 0.18 mm. The effect was “moderate” when a year-change in refraction ranged from 0.25D to 0.50D or in axial length ranged from 0.09 mm to 0.18 mm, and the effect was “weak” when one year-change in refraction ranged from 0 to 0.25D or in axial length from 0 mm to 0.09 mm,

## Result

### Search results and study characteristics

**Fig. 1:**
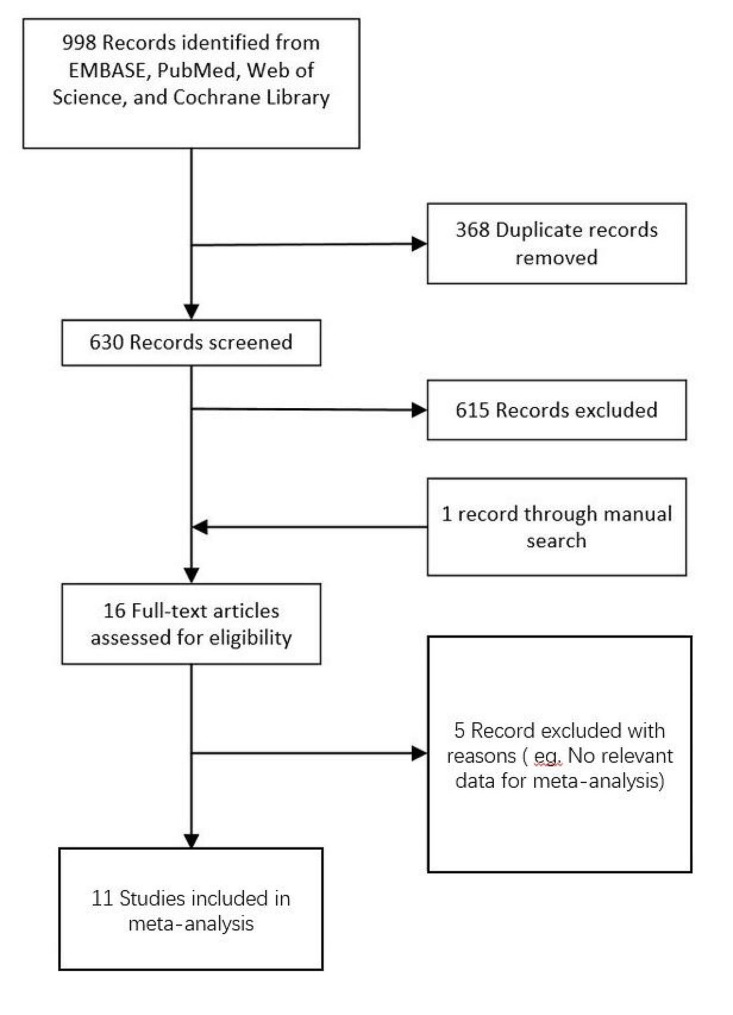
Flow chart of the literature search and study selection

The process of selecting studies that were included in our review is outlined in Fig. 1. Initially, 998 studies were identified through a comprehensive electronic literature search. After screening references, 11 studies met the predetermined inclusion and exclusion criteria, Which consisted of 8 RCTs (Fujikado et al. 2014; Cheng et al. 2016; Jones et al. 2022; Raffa et al. 2020; Valle et al. 2020; Walline et al. 2020; Sankaridurg et al. 2018; Jianxia Fang et al. 2022) and three prospective cohort studies (Walline et al. 2013; Sankaridurg et al. 2011; Paune et al. 2015). The evaluation of peripheral add multifocal SCLS was the focus of all 11 studies, with five of them examining peripheral gradient SCLs [6, 11–14], five examining central-distance multifocal SCLs [15–19], and one examining positive spherical aberration SCLs [20]. These studies involved a total of 787 patients: 393 patients were in the peripheral add multifocal SCLs group, and 394 were in the control group. Table 1 summarizes the key characteristics of the included studies. The risk of bias for each study was assessed independently by two authors (Tables 2 and 3).

**Table 1:** Characteristics of the studies included in the meta-analysis

Study	Design	Follow up (months)	Age (years)	plus power	Subjects (E/C)	Treatment lens	Baseline refraction(D)	Treatment effect	
								Axial length	Myopia control
Cheng USA, 2019	RCT	12	8–11	+ 0.175 μm	53/59	Positive spherical aberration	-2.25 ± 1.09	37%	18%
Fujikado Japan,2014	RCT	12	10–16	+ 0.50D	24/24	Menicon low-addition	−0.75 ∼ − 3.50	47%	26%
Sankaridurg China, 2019	RCT	24	8–13	+ 1.50D	101/102	Progressive periphery	−0.75 ∼ − 3.50	23%	21%
Walline USA, 2020	RCT	24	7–11	+ 2.50D	97/97	multifocal soft lenses	−1.00 ∼ − 6.00	36%	43%
Garcia-del Valle Spain,2020	RCT	12	7–15	+ 2.00D	36/34	progressive multifocal and reverse geometry	-0.50∼-8.75	41%	51%
Lina H. Raffa, Malaysia, 2022	RCT	18	13–15	+ 3.00	9/10	Proclear multifocal contact lenses	-2.00∼-6.00	63%	66.6%
Jenny Huang Jones, USA, 2022	RCT	36	7–11	+ 2.50D	46/46	Biofinity Multifocal “D” contact lenses	-0.75∼-5.00	43%	50%
Jeffrey J Walline, USA, 2013	prospective, cohort study	24	8–11	+ 2.00	32/32	Proclear Multifocal “D”	-1.00∼-6.00	50%	29%
Padmaja Sankaridurg, China, 2011	prospective, cohort study	12	7–14	+ 2.00	40/40	novel contact lenses	-0.75∼-3.50	34%	33%
Jaime Pauné, Spain, 2015	prospective, longitudinal, nonrandomized study	24	9–16	+ 6.00	19/21	soft radial refractive gradient (SRRG) contact lenses	-0.75∼-7.00	43%	27%
Jianxia Fang, china, 2022	RCT	12	7–15	+ 6.00	22/24	The BioThin (Bio Optic, Inc., Taiwan, China) MFSCLs	-1.00∼-8.00	26.8%	37%

E = Experiment group; C = Control group; SCLs = Single vision contact lens; RCT = randomized controlled trials

**Table 2:** Quality assessment of RCTs

Evaluation Metrics	Cheng 2019	Fujikado 2014	Snkaridurg 2019	Walline 2020	Valle 2020	Lina 2022	Jenny 2022	Fang 2022
Random sequence generation	L	L	L	L	L	L	L	L
Allocation concealment	L	U	L	L	L	L	L	L
Blinding of participants and personnel	L	L	L	L	L	L	L	H
Blinding of outcome assessment	L	L	L	L	L	L	L	L
Incomplete outcome data	L	L	L	L	L	L	L	L
Selective reporting	L	L	L	L	L	L	L	L
Other bias	U	U	U	U	U	U	U	U

L = low; U = unclear; H = high

**Table 3:** Quality assessment of cohort studies

Evaluation Metrics	Jaime 2015	Padmaja Sankaridurg 2011	Jeffrey J Walline 2013
Was the cohort recruited in an acceptable way (robust inclusion/exclusion criteria or consecutive recruitment)	Y	Y	Y
Was the study prospective?	Y	Y	Y
Was the intervention conducted in an explicit and standardised manner (e.g., guidelines/protocol applied)	Y	Y	Y
Was the outcome appropriately measured to minimise bias?	N	N	N
Did they identify important confounding factors (e.g., age at intervention, baseline angle of deviation)	Y	Y	Y
Did they adjust for confounding factors in the design and/or analysis where necessary	Y	Y	Y
Were they followed-up for at least 12 months?	Y	Y	Y
Are the authors’ conclusions substantiated by the reported data?	Y	Y	Y

### Refraction

**Fig. 2:**
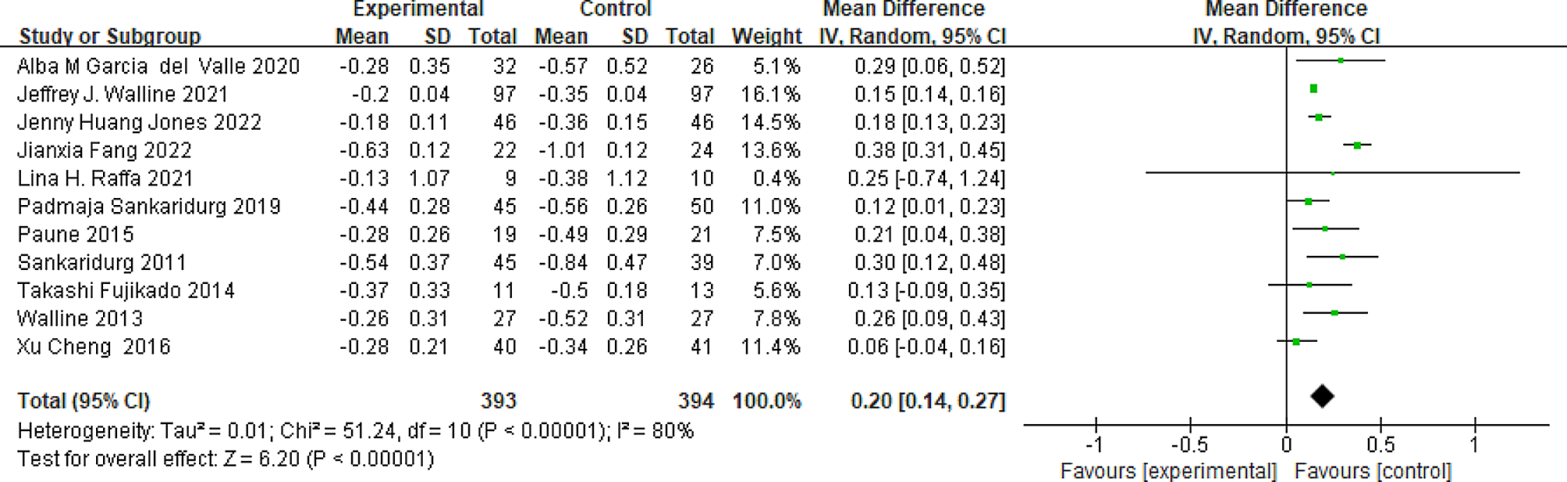
Peripheral defocus modifying contact lenses vs. the control group on the change in myopia progression. CI = confidence interval

Data were obtainable from a total of 11 studies. The graphical representation in Fig. 2 demonstrated that peripheral add multifocal SCLs groups exhibited a significantly reduced progression in refraction compared to the control group (MD = 0.20; 95%CI, 0.14 ∼ 0.27; *P*<0.001; Fig. 2). There was significant heterogeneity observed among the included studies (*P*<0.01, I^2^ = 88%). Notably, upon the exclusion of the study conducted by Fang et al., the heterogeneity decreased from 88 to 15%, and the overall effect remained consistent, as depicted in Fig. 2.

### Axial elongation

**Fig. 3:**
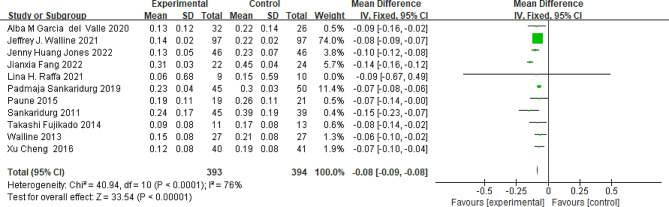
Peripheral defocus modifying contact lenses vs. the control group on the change in axial elongation. CI = confidence interval

All the included studies reported changes in axial length. The pooled data revealed a statistically significant reduction in axial length elongation in the peripheral add multifocal SCLs group, compared to the control group (MD=-0.08; 95%CI, -0.09∼-0.08; *P*<0.001; Fig. 3). There was significant heterogeneity observed among the included studies (*P*<0.01, I^2^ = 76%). Upon exclusion of Fang et al.‘s study, the heterogeneity decreased from 76 to 0%, while the overall effect remained unchange (Fig. 3).

### Visual acuity

**Fig. 4:**
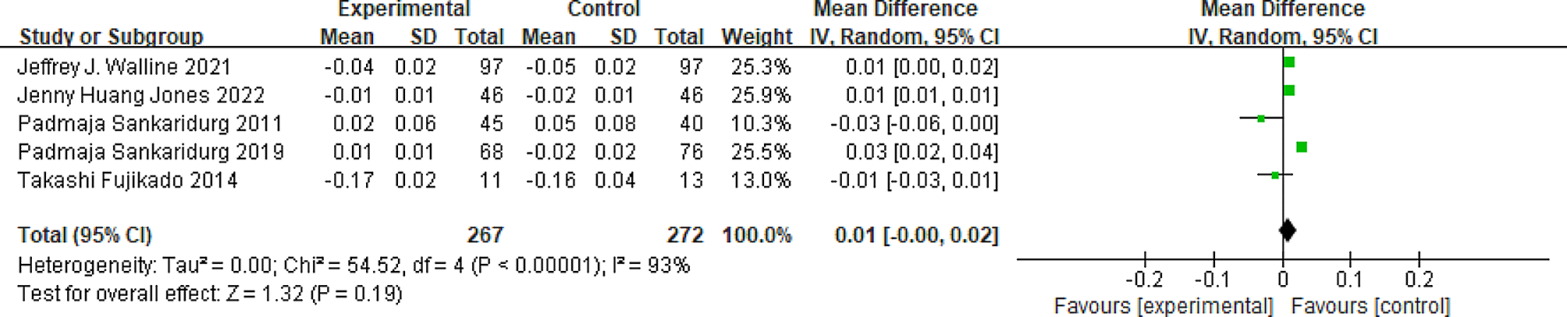
Peripheral defocus modifying contact lenses vs. the control group in High-contrast visual acuity. CI = confidence interval

Five studies included in the analysis reported data on high-contrast visual acuity measured in LogMAR units. The results showed no significant difference in high-contrast distance logMAR visual acuity between the peripheral add multifocal SCLs group and the single vision group (MD = 0.01; 95%CI, -0.00 ∼ 0.02; *P* = 0.19; Fig. 4). However, the included studies exhibited significant heterogeneity(*P*<0.01, I^2^ = 93%).

**Fig. 5:**
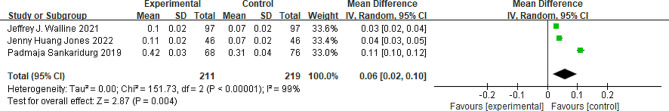
Peripheral defocus modifying contact lenses vs. the control group in Low-contrast visual acuity. CI = confidence interval

Three studies presented data on low-contrast visual acuity measured in LogMAR units. The group using peripheral add multifocal SCLs showed statistically significant worse results than the single vision group (MD = 0.06; 95%CI, 0.02 ∼ 0.10; *P* = 0.004; Fig. 5). There was significant heterogeneity across studies (*P*<0.01, I^2^ = 99%).

### Adverse effects

**Fig. 6:**
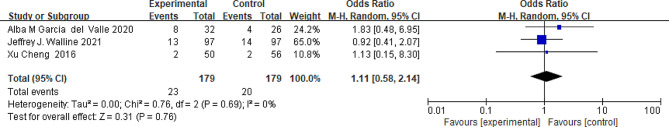
The contact lens related adverse events for SMCLs groups versus controls. CI = confidence interval

Three studies (involving 461 patients) examined adverse effects (AEs) related to soft contact lens wear. In total, there were 46 lens-related adverse events, all of which were categorized as highly likely or possibly/probably related to the contact lenses. None of these events were severe enough to warrant permanent discontinuation of contact lens use. The overall incidence of lens-related adverse events was calculated to be 9.98%. A random-effects model was employed to synthesize the data, revealing an incidence rate of 0.065 (0.048 ∼ 0.083) for adverse events associated with contact lens wear. The combined results indicated an OR of 1.11 (95%CI, 0.58 to 2.14; *P* = 0.76) between the defocus SCLs group and the single vision SCL group. No statistically significant differences in the incidence rates of all adverse events were observed between the two groups, as shown in Fig. 6. Additionally, when all infiltrative events were aggregated (including eight asymptomatic events), the crude incidence was calculated to be 1.80%. Lastly, it is worth noting that only Jeffrey’s study reported a single case of probable microbial keratitis.

### Subgroup analysis

A subgroup analysis was performed according to the comparison groups (single vision SCLs or spectacles). 3 studies used single vision spectacles as the control group, while the remaining 8 studies used single vision SCLs. The results exhibited that differences between subgroups demonstrated statistical significance(*P* = 0.002 and 0.03). Peripheral add multifocal SCLs showed better results in delaying myopia and eye axis prolongation when choosing single vision spectacles as the control group.

### Sensitivity analysis and publication bias

Sensitivity analyses by excluding each study did not yield significant alterations in the respective results, affirming the robustness and reliability of the findings. Egger’s test showed that there was no publication bias (*P* > 0.05).

### Grading the quality of evidence

GRADEpro was unable to simultaneously evaluate the quality of cumulative evidence from both randomized controlled trials and observational trials. Therefore, it may be beneficial to assess the quality separately. This meta-analysis included 8 high-quality RCTs. No high risk of bias was identified in any of the datasets (outcomes). There were no concerns of indirectness in any of the datasets (outcomes). However, there was considerable heterogeneity (high inconsistency) across all datasets (outcomes), leading to a downgrade in the quality of all pooled mean differences (MD) by one level. The low-contrast visual acuity dataset had wide confidence intervals (imprecision), resulting in the downgrade of the pooled MD by one level. Consequently, the quality of the pooled MD for low-contrast visual acuity was downgraded by two levels, from high to low. For the pooled MD of the other outcomes, each estimate’s quality was downgraded by one level, from high to moderate.

Additionally, three high-quality prospective, longitudinal, nonrandomized studies were included in the meta-analysis. Due to the small number of studies, only two main indicators (refraction and axial length) were assessed. The large effect value observed in all datasets (outcomes) led to an upgrade in the quality of all pooled MD by one level. Therefore, the quality evaluation results for observation studies was consistent with randomized controlled trials, and the evidence was graded as moderate quality.

## Discussion

An investigation was conducted to analyze the impact of peripheral add multifocal SCLs on the deceleration of myopia progression. This analysis included eight published RCTs and three cohort studies. The combined findings indicated that peripheral add multifocal SCLs were more effective in reducing refractive progression and axial growth compared to single vision SCLs or spectacles. Peripheral add multifocal SCLs did not affect high-contrast distance logMAR visual acuity but resulted in a significant decrease in low-contrast logMAR visual acuity at distance. The incidence of adverse effects related to soft contact lenses was low, and there was no significant difference in the frequency of all reported adverse events between peripheral add multifocal SCLs and single vision SCLs.

In comparison to the control group, peripheral add multifocal SCLs exhibited promising outcomes in controlling myopia among school-aged children, with effects sizes of 0.20D in decelerating myopia progression and 0.08 mm in reducing axial elongation, respectively. These values were marginally smaller than those reported in other studies involving orthokeratology and atropine. Huang et al.‘s study revealed that atropine eye drops had a significant effect in controlling myopia, while orthokeratology and SCLs with peripheral defocus design exhibited moderate effects [21]. It should be noted that Huang et al.‘s meta-analysis only included three studies that compared peripheral defocus modifying contact lenses with single vision contact lenses. Consistent with our findings, peripheral add multifocal SCLs exhibited a weaker effect in controlling myopia. For practitioners dealing with a child who requires myopia control, it is recommended to consider the use of orthokeratology lenses or low-concentration atropine. In certain cases, multifocal soft contact lenses could be recommended: (1) Individuals with low myopia: multifocal soft lenses provide a stable and significant defocus effect that fulfills the requirements for myopia control. (2) Patients with a flat cornea, a small e-value of corneal topography, and a higher degree of myopic refraction, and those who are unable to achieve optimal vision with orthokeratology lenses due to insufficient sleep time at night, can benefit from multifocal soft lenses that address both vision improvement and myopia control needs. (3) Myopia occurs at a younger age. (4) Patients with mild trichiasis: wearing SCLs during the day can protect the cornea from mechanical damage caused by trichiasis.

Significant heterogeneity was observed when comparing the efficacy of peripheral add multifocal SCLs with single-vision SCLs or spectacles. Heterogeneity persisted in the random-effects model, prompting a sensitivity analysis to identify its sources. The study conducted by Fang et al. was identified as the primary contributor to this heterogeneity [13]. Compared with other multifocal SCLs, the multifocal SCLs tested in this study differed in optimal design, defocus amount, and size of the central distance zone. In Fang et al.’s study, the defocus amount of the MFSCLs was + 6.00 D [13], compared to + 2.00D reported by Anstice et al. [22], + 1.00 D reported by Sankaridurg et al. [6], and + 2.50 D reported by Lam et al. [23], which wouldimpact the ability of multifocal SCLs to control myopia progression.

This meta-analysis also investigated the impact of peripheral add multifocal SCLs on visual performance, focusing on high- and low-contrast visual acuity. The study revealed that defocus SCLs did not affect high-contrast distance logMAR visual acuity, but caused a significant decrease in low-contrast logMAR visual acuity at distance. Visual performance with peripheral add multifocal SCLs was slightly worse at nighttime compared to daytime. Kang et al. discovered that the greater the progressive-addition power, the more noticeable the decline in low-contrast VA [24]. The peripheral add multifocal SCLs have a design with a central zone for distance correction and peripheral near addition zones that cause myopic defocus of the peripheral retina. However, this design also increases positive spherical aberration which affects central vision more in young people due to their larger natural pupils [25]. The relatively small central area of the peripheral add multifocal SCLs ensures multifocality in the pupillary region at most distances in most young subjects, but this also results in reduced visual quality compared to SV lenses. Specially designed multifocal SCLs may lead to decreased image quality and associated visual symptoms, particularly in low illumination and contrast conditions [26]. Kang et al. assumed that high-contrast VA is not a very sensitive measure of visual quality. Future clinical trials testing the efficacy of multifocal lenses for myopia control should consider low-contrast VA to better characterize the effects on vision of such lenses and allow for more informed comparison of alternative treatment options. When prescribing multifocal SCLs for myopia control, clinicians should educate patients about these effects on vision.

Another concerning aspect is ocular adverse events associated with the use of soft contact lenses. A recent review paper concluded that the occurrence of corneal infiltrative events does not seem to be higher in children than in adults [27]. Chamberlain et al. reported a total of 18 ocular adverse events (involving 11 subjects) in individuals wearing the MiSight lens, while 12 events (involving ten subjects) were observed in those using the control lens after three years of treatment. Among these, seven events (12.5%) with the test lens and seven events (13.2%) with the control lens were deemed lens-related. These complications were mostly mild in nature and did not require any therapeutic intervention [5]. Cheng et al.‘s study reported a total of 6 mild ocular AEs in three subjects (1.6%) during the course of treatment and withdrawal phase. Among these, four events were observed in four eyes of two subjects in the test cohort (allergic conjunctivitis), and the remaining event occurred in two eyes of one subject in the control cohort (contact dermatitis). All AEs were classified as nonsignificant and were considered unlikely to be associated with wearing the study contact lenses [20]. In Walline et al.‘s study, a total of thirty-five ocular adverse events (12%) that were definitely or probably related to contact lens wear were of moderate severity. None of the reported ocular adverse events were deemed serious, severe, or resulted in permanent discontinuation of contact lens wear [17]. In our study, out of the 461 individuals, 46 patients experienced lens-related complications, resulting in a combined incidence rate of 6.5%. None of these complications were severe. Allergic conjunctivitis and unspecified conjunctivitis were the most frequently observed complications, which did not require specific management. Therefore, this study highlights peripheral defocus modifying contact lenses as a comparatively safe treatment. It is important to note that the findings of the analyzed studies were obtained under controlled and optimal conditions. In real-life situations with a large number of cases and potentially reduced supervision and care, there is likely to be an increase in side effects and complications. If patients want to control myopia by wearing multifocal SCLs, it can be emphasized that SLCs are a safer option with lower and milder adverse reactions. This can help reduce the patient’s psychological burden and minimize the rate of drop out, ultimately improving the effectiveness of myopia control.

Therapy adherence is a significant concern that requires attention. In the study conducted by Walline et al. [15], the dropout rate was reported to be 33%. Lam et al. reported a dropout rate of 41% [23], Chamberlain et al. reported a rate as high as 43% [5]. Sankaridurg et al. reported a rate of 25% [11], and Paune et al. reported a rate of 37% [12]. Sankaridurg et al. reported a large number of children discontinued soon after lens dispensing (129/ 508, 25.4%) and prior to the 1 month visit. The main reasons for dropping out were: discomfort with lens wear (26/ 129, 20.2%); safety concern with contact lenses (25/129, 19.4%); no interest in contact lens wear (25/129, 19.4%); handling (15/129 or 11.6%); time conflicts and issues with attending follow up (10/129 or 7.8%); and other reasons such as red eye, rhinitis, preferred orthokeratology and unable to attend due to relocation. Of the remaining participants that continued to wear lenses, 89 participants (23.5%) were discontinued/lost to follow up over 2 years. The main reasons were: discomfort (19/89, 21%); time conflicts (12/89, 14%); lost to follow (10/89, 11%); handling (8/89, 9%); increased myopia progression (9/89, 10%); vision problems with lenses (2/89, 2%); and prefer to switch to orthokeratology (3/89, 3%) [6]. During the entire course of the treatment phase in Xu Cheng et al.’s study, a total of 14 (22%) subjects from the test SCLs cohort were discontinued from the study. Subject disinterest(3)、noncompliance to protocol(2)、 lens handling difficulties(4)、lens discomfort(3) and lost to follow-up(2) were reported as the main reasons [20]. Sankaridurg et al. also discovered discomfort was the most frequently cited reason for discontinuation from lens wear (11.7%), followed by handling issues (1.7%). Noncontact lens–related reasons such as geographic relocation (8.3%) and disinterest (6.7%) were substantial [11]. In Chamberlain et al.’s study, 12 participants (18%) from the test SCLs group discontinued the intervention program. Lens related reasons were discomfort, vision and subjects or parents/guardian decision(*n* = 5). Non-lens related reasons were loss of motivation, schedule, diagnosed diabetic, and protocol violation(*n* = 5) [5].

It can be seen that discomfort and lens handling difficulties were the most common causes of dropout. Contact lens fitters should provide patients and their parents with a comprehensive explanation regarding the possibility of discontinuing the use of multifocal SCLs prematurely, primarily due to discomfort or difficulties in handling. Another common reason for dropout was time conflicts. Initially, many participants expressed a desire to wear contact lenses as a means to slow down the progression of myopia. They were unwilling to wear lenses every day afterwards because they were too busy with school curriculum and homework, and too rushed to wear lens in early morning. Medical staff should inform patients in advance that it is advisable to avoid wearing multifocal SCLs if the patient has time pressure in the morning or is unable to consistently wear them. The study showed that only a small number of patients experienced complications, indicating that multifocal SCLs may be a safer choice. Clinicians can emphasize this finding when recommending contact lenses to patients.

Li et al. conducted a meta-analysis to evaluate the impact of concentric ring bifocal and peripheral add multifocal SCLs on the retardation of axial length growth [7]. However, certain limitations were present in their study. Firstly, the primary measure of treatment outcome was the increase in refraction and axial length in the first year, which may not accurately reflect the true status of myopia progression. Secondly, reliable conclusions cannot be drawn solely based on this meta-analysis, as it included only 8 studies, of which only 5 were RCTs. Thirdly, Li et al. did not examine the visual performance and safety of soft contact lenses. In our study, 11 high-quality studies were included and the mean annual change in refraction and axial length were chosen as the main comparative indicators. Fourthly, to reduce heterogeneity, bifocal SCLs were not included in our meta-analysis. Additionally, the visual performance and adverse effects associated with soft contact lenses were evaluated.

In this meta-analysis, three studies used single vision spectacles as the control group, while the remaining 8 studies employed soft contact lenses. The results of the subgroup analyses showed that peripheral add multifocal SCLs showed better results in delaying myopia and axis length prolongation when choosing single vision spectacles as the control group. Following the recommendation of the Food and Drug Administration Public Workshop on Controlling the Progression of Myopia [28], it is more appropriate to select the latter as a control. The lens was well-matched in all parameters, except for the peripheral add multifocal soft contact lenses, which ensures that any disparities in refraction and axial length between the two groups can be attributed to the optical design. In our meta-analysis, multifocal SCLs demonstrated superior effects in slowing refraction progression and axial growth compared to the control group. These findings remained consistent even after excluding studies that used single vision spectacles as control. It is this distinctive design that played a pivotal role in controlling myopia progression.

Among the studies included in this article, 5 articles included patients with myopia ranging from − 0.75D [6, 11, 12, 16, 18], 3 articles starting from − 1.00D [13, 15, 17], 1 article starting from − 2.00D [19], and another article starting from − 0.50D [14]. It can be observed that there is no consensus on the starting power of soft contact lenses for myopia correction. Typically, myopia of 0.75 ∼ 1.00D requires the use of glasses to improve vision. We believe that myopia of 0.75 ∼ 1.00D should be considered as a reasonable starting point for multifocal SCLs. However, one document in this study used a lower starting degree of myopia range (-0.50D), and the author explained that the inclusion criteria range for refraction(-0.50 to -8.75) was set to facilitate subject recruitment. In clinical practice, the initial correction power can be appropriately reduced (such as -0.50D) for the myopic patients with the goal of controlling myopia: Children and adolescents with myopic progression ≥ 0.50 D/year or axial elongation ≥ 0.4 mm/year; Patients with fundus pathological changes associated with axial elongation; patients with high-risk factors for myopia and/or high myopia, such as parents with a history of myopia, less time spent on outdoor activities, prolonged near work, myopia occurring in children at an early school age (7 years old and under), and myopia being higher than that of children and adolescents of the same age. One reason is that multifocal SCLs have both a stable and relatively high defocus amount, which can meet the defocus requirements for myopia control. The other reason is that their clinical side effects are relatively small and controllable.

Our study also had certain limitations and drawbacks. Firstly, the available pool of subjects included in this meta-analysis was limited, albeit being the largest reported thus far. Secondly, the studies included in this analysis had different add power for the multifocal SCLs. Thirdly, the quality of the conducted studies varied, with some lacking a double-blinded design. Fourthly both single vision SCLs and spectacle lenses were selected as control measures. Fifthly, there was an age disparity among the studies. The included articles only provided the age range and mean, which was insufficient to explore the relationship between therapeutic outcomes and age. Lastly, significant heterogeneity was observed in certain pooled analyses.

In conclusion, the findings of this meta-analysis indicate peripheral add multifocal SCLs were effective in slowing down myopia progression. However, the effect of this kind of lenses was weaker and may also lead to a significant decrease in distance low-contrast logMAR visual acuity. Despite this, the study found that SCLs were a safe treatment option with a lower incidence of adverse effects and no serious complications. Further research is needed to address several issues. For instance, the lack of a uniform add powers poses a challenge. In our meta-analysis, the included studies employed different plus power for multifocal SCLs, highlighting the need for further investigation to determine the optimal myopic defocus amount for achieving the desired myopia control effect. Specifically, more studies should be conducted to ascertain the long-term efficacy of multifocal SCLs in slowing down the progression of myopia, establish the optimal duration of SCLs wear to achieve an optimal therapeutic effect, and evaluate the impact of discontinuing long-term lens wear on the subsequent progression of myopia.

### Electronic supplementary material

Below is the link to the electronic supplementary material.

Supplementary Material 1

## Data Availability

Data used in the analyses can be found in the published article, which were listed in the references of this manuscript.

## Abbreviations

SCLs: soft contact lenses
SVSCLs: single-vision soft contact lenses
RCT: randomized controlled trials
MD: mean difference
OR: Odds ratio
